# Are serum levels of inhibin A in second trimester predictors of adverse pregnancy outcome?

**DOI:** 10.1371/journal.pone.0232634

**Published:** 2020-05-29

**Authors:** Chao-Yan Yue, Chun-Yi Zhang, Ying-Hua Ni, Chun-Mei Ying

**Affiliations:** Department of Laboratory Medicine, Obstetrics and Gynecology Hospital of Fudan University, Shanghai, China; University of Mississippi Medical Center, UNITED STATES

## Abstract

**Objective:**

During pregnancy, inhibin A is mainly derived from the placenta and regulates the implantation and differentiation of embryos. Our aim was to assess whether second trimester serum inhibin A was associated with an increased risk of adverse pregnancy outcomes.

**Methods:**

We investigated the serum levels of Inhibin A during the second trimester in pregnancy, and analyzed associations between the Inhibin A and the risk of adverse pregnancy outcome. 12,124 pregnant women were enrolled in this study between January 2017 and July 2019 at the Obstetrics & Gynecology Hospital of Fudan University. Multivariate logistic regression analysis was conducted to estimate the relative risk between Inhibin A and adverse pregnancy outcome.

**Results:**

Compared with the group without adverse pregnancy outcome, during the second trimester of pregnancy, age and Inhibin A were risk factors for pre-eclampsia, gestational diabetes mellitus and preterm delivery; Inhibin A was risk factors for low birth weight. Gravidity and Inhibin A were risk factors for macrosomia; while parity was a protective factor against pre-eclampsia, gestational hypertension and low birth weight.

**Conclusion:**

Elevated Inhibin A levels in pregnancy are significantly associated with pre-eclampsia, GDM, macrosomia, low birth weight and preterm delivery.

## 1. Introduction

Inhibin, a glycoprotein dimer hormone linked by disulfide bonds, is a member of the transforming growth factor-b superfamily, composed of α-subunit and one of several β-subunits. Including inhibin A (α-βA) and inhibin B (α-βB) [1]. During pregnancy, inhibin A is mainly synthesized and secreted by placental syncytiotrophoblasts, which is involved in the regulation of various hormones in the placental local regulatory axis, and is related to endometrial decidualization, embryo implantation, proliferation and differentiation of trophoblasts. It affects the occurrence, development and maintenance of pregnancy. Therefore, inhibin A can affect fetal growth and pregnancy outcome. The level of inhibin A reached the first peak at 8–10 weeks of pregnancy, was relatively stable at 14–30 weeks, increased gradually from the third trimester of pregnancy to full term, and reached the highest level at delivery [2–5].

Previous studies have reported that the increase of inhibin A is associated with the occurrence of preeclampsia, and fetal malformation and fetal growth restriction [2, 6–10], but there are few reports on inhibin A and other maternal and fetal adverse pregnancy outcomes. The purpose of our study was to evaluate the relationship between inhibition of A and preeclampsia, gestational hypertension, gestational diabetes, macrosomia, low birth weight and preterm delivery in Chinese pregnant women.

## 2. Methods

### 2.1 Subjects

A total of 12,124 singleton pregnancies women with a live delivery between January 2017 and July 2019 at the Obstetrics & Gynecology Hospital of Fudan University (Shanghai, China) were enrolled in this study. Women without complications are controls. The clinical data and outcomes for mothers and neonates were obtained from clinical records.

Pre-eclampsia was diagnosed at weeks 20 through 39 of gestation, using the current American College of Obstetricians and Gynecologists (ACOG) guidelines [11]. Gestational hypertension is defined as diastolic blood pressure > 90 mmHg on two occasions four hours apart, or > 110 mmHg once, or systolic blood pressure > 140 mmHg on two occasions four hours apart, or > 160 mmHg once, after 20 weeks’ gestation (or a combination) [12]. GDM was diagnosed at 24–28 weeks of gestation using the American Diabetes Association (ADA) criteria [13]. Macrosomia was defined as birth weight >4000g. Low birth weight was defined as birth weight less than 2500 g. Preterm birth was defined as gestational age at birth of less than 37weeks. Preterm delivery includes spontaneous preterm labor, preterm prematurely ruptured membranes and preterm birth for medical and obstetrical indications.

Inclusion criteria: singleton pregnant women who underwent Down's screening and delivery in our hospital. Study exclusion criteria were as follows: established type 1 or type 2 diabetes; established hyperlipidemia, hypertension, cardiovascular diseases or metabolic syndrome before pregnancy; a history of severe systemic disease such as liver cirrhosis, chronic renal failure, severe anemia or immune disorders; and untreated endocrinopathies (hyperadrenalism, hypoadrenalism, and hyperthyroidism or hypothyroidism), or patient had no complete maternal and infant record.

The present study conformed to the principles of the Declaration of Helsinki. Approval was obtained from the Research Ethics Committee of the Obstetrics & Gynecology Hospital of Fudan University (approval number: 2019–06), and written consent was obtained from all women in this study.

### 2.2 Serum samples

Five milliliters of peripheral blood were collected from fasting participants between weeks 14 and 20 of gestation. The peripheral blood was collected in a serum separator tube and samples were allowed to clot for 30 minutes before centrifugation at 1000 × *g* for 5 min. All peripheral blood samples were processed within 2 h of collection.

### 2.3 Biochemical analyses

Down's screening is a routine prenatal examination. Down's screening in our hospital is a serological quadruple screening, including inhibin A. Serum samples were analyzed for Inhibin A using Beckman Coulter instruments. The analytical performance of the measurements assessed with control materials showed values within the recommended limits. Interassay coefficients of variation (CV%) were less than 10%.

We measured the level of inhibin A in the serum of pregnant women, converted the measured value to a multiple of the median (MoM), and corrected it according to the mother's age, body mass, smoking status, gestational age and whether there was insulin dependent diabetes mellitus.

### 2.4 Statistical analysis

All statistical analyses were performed using GraphPad Prism version 5.0 for Windows (GraphPad Software, USA). The normal distribution data are expressed as mean ± SD. Non-normal distribution data are expressed as median and percentiles (Q1, Q3). Associations between Inhibin A and the risk of adverse pregnancy outcome were tested by multivariate logistic regression analysis. Variable selection in multivariable modeling was based on clinical and statistical significance. P < 0.05 was considered a statistically significant difference.

## Results

Of the 12,124 pregnant women we observed from January 2017 to July 2019. Participants included 8333 normal pregnancies in women and pregnancies in women with adverse pregnancy outcome were divided into six groups: pre-eclampsia (n = 560), Gestational hypertension (n = 505), gestational diabetes mellitus (GDM) (n = 1336), macrosomia (n = 699), low birth weight (n = 276) and preterm delivery (n = 415). The incidence of preeclampsia, gestational hypertension and gestational diabetes was 4.62%, 4.17% and 11.02%, respectively. The incidence of macrosomia, low birth weight and premature delivery was 5.77%, 2.28% and 3.42%, respectively.

All patient characteristics, including Inhibin A and Inhibin A MoM are described in Table 1.

**Table 1:** Characteristics of mothers and infants.

	preeclampsia	Gestational hypertension	GDM	macrosomia	low birth weight	preterm delivery	Unaffected
	n = 560	n = 505	n = 1336	n = 699	n = 276	n = 415	n = 8333
maternal							
Age (years)	29.3±2.72	29.5±2.82**	29.9±2.76***	29.3±2.96	29.3±2.82	29.6±2.68***	29.0±2.82
Pre-pregnancy BMI	22.9±3.81***	23.3±3.93***	22.5±3.53***	22.5±3.31***	21.2±3.02***	21.6±3.27***	20.7±2.58
Parity	1.13±0.35***	1.15±0.363*	1.24±0.45	1.29±0.49*	1.14±0.36	1.22±0.46	1.22±0.44
Gravidity	1.47±0.81**	1.53±0.806	1.67±0.95*	1.73±1***	1.5±0.84	1.61±0.92	1.58±0.88
Log_10_ Inh A	2.34(164,304)***	2.30±0.20	2.31±0.19	2.30±0.17	2.35±0.25***	2.33±0.22**	2.30±0.17
Inh A MOM	1.34±0.83***	1.15±0.56	1.13±0.54**	1.15±0.48***	1.4±0.98***	1.29±0.83***	1.07±0.47
Detection of gestational age	16.6±0.97	16.7±1.00	16.7±1.03	16.9±1.04	16.6±0.93	16.7±0.99	16.7±1.00
Infant							
Gestational age weeks	38.2±1.73***	39.0±1.26	38.7±1.27***	39.7±0.904***	35.3±2.71***	34.8±1.74***	39.1±1.06
Birth weight (g)	3172±603***	3353±434	3349±469	4214±198***	2137±363***	2517±540***	3339±326
Apgar score	8.86±0.78***	8.87±0.84	8.93±0.49	8.84±0.88***	8.48±1.71***	8.52±1.69***	8.95±0.49

*P < 0.05

**P < 0.01,

***P < 0.001, compared with Unaffected. GDM: Gestational diabetes mellitus, BMI: Body mass index, MOM: multiple of the median. The normal distribution data are expressed as mean ± SD. Non-normal distribution data Inh A are expressed as median and percentiles (Q1, Q3) after log10 transformation.

Factors with significant differences from the univariate analysis were included in the regression analysis. Multifactorial logistic regression analysis of each procedure was conducted based on univariate analyses. Regression analysis was used to adjust the influence of confounding factors, including age, parity, gravidity and pre-pregnancy BMI. The MoMs of Inh A have been log transformed in the logistic regression analyses.

After adjustment for all confounding factors, Inhibin A was risk factors for preeclampsia (OR = 7.453), gestational hypertension(OR = 1.442), gestational diabetes(OR = 1.458), macrosomia(OR = 2.217), low birth weight (OR = 7.241), preterm delivery (OR = 4.765); age was risk factors for preeclampsia (OR = 1.034), gestational hypertension(OR = 1.056), gestational diabetes(OR = 1.113), preterm delivery (OR = 1.057); while parity was a protective factor against pre-eclampsia(OR = 0.551), gestational hypertension (OR = 0.599) and low birth weight(OR = 0.653), these differences were statistically significant (P < 0.05) (Table 2).

**Table 2:** Multivariate regression analysis of inhibin A and adverse pregnancy outcome.

	β	S.E.	WaldS	*P*	adjusted Odds ratio	95% CL lower	95% CL upper
PE							
Age	0.033	0.016	4.077	0.043	1.034	1.001	1.068
Parity	-0.597	0.128	21.641	0.000	0.551	0.428	0.708
Inh A MOM	2.009	0.237	71.978	0.000	7.453	4.686	11.855
Gestational hypertension							
Age	0.055	0.017	10.102	0.001	1.056	1.021	1.093
Parity	-0.512	0.126	16.499	0.000	0.599	0.468	0.767
Inh A MOM	0.366	0.261	1.977	0.160	1.442	0.866	2.404
GDM							
Age	0.107	0.011	98.601	0.000	1.113	1.089	1.136
Inh A MOM	0.377	0.167	5.125	0.024	1.458	1.052	2.020
macrosomia							
Gravidity	0.182	0.039	21.933	0.000	1.200	1.112	1.295
Inh A MOM	0.796	0.221	12.975	0.000	2.217	1.437	3.418
low birth weight							
Parity	-0.427	0.168	6.430	0.011	0.653	0.469	0.908
Inh A MOM	1.980	0.333	35.394	0.000	7.241	3.772	13.903
preterm delivery							
Age	0.055	0.018	9.218	0.002	1.057	1.020	1.095
Inh A MOM	1.561	0.277	31.795	0.000	4.765	2.769	8.199

The MoMs of Inh A have been log transformed in the logistic regression analyses.

**Fig 1:**
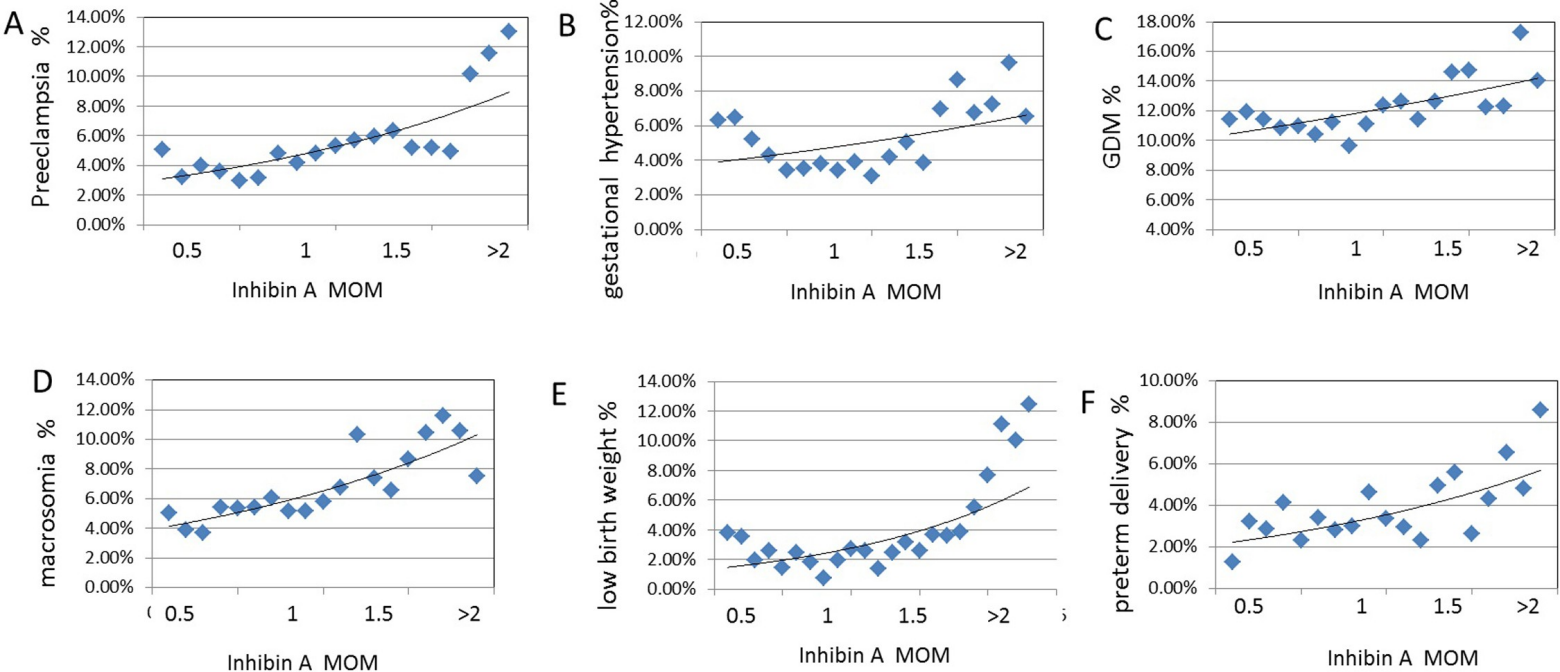
Incidence of adverse pregnancy outcomes at different inhibin A MOM values. The blue dots were incidence in the population divided into 19 groups according to inhibin A MOM.

In Fig 1, the blue dots were incidence in the population divided into 19 groups according to inhibin A MoM. We can observe that the incidence of adverse pregnancy outcome varies greatly with the moment value of inhibin A. When inhibin A MoM exceeded 2, the incidence of adverse pregnancy outcomes of these mothers and fetuses increased significantly (Fig 1).

## Discussion

Preeclampsia, gestational hypertension, gestational diabetes, preterm delivery, macrosomia and low birth weight are common obstetric complications that endanger maternal and infant health. Our study focused on serum inhibin A levels from 14 to 20 weeks of pregnancy and evaluated their relationship with these pregnancy complications. The results showed that serum inhibin A was an independent risk factor for preeclampsia, gestational hypertension, gestational diabetes, macrosomia, low birth weight and preterm delivery in Chinese pregnant women.

There is no consensus on the relationship between inhibin A and hypertensive disorder complicating pregnancy (including preeclampsia and hypertensive disorder complicating pregnancy). Although D'Anna R et al. and Tul et al. points out that inhibin A is not associated with preeclampsia [14, 15], more studies suggest that elevated inhibin A is involved in the occurrence of preeclampsia [2, 7, 10, 16, 17], and the concentration of inhibin A is closely related to the severity of preeclampsia [18, 19]. Akolekar R et al. and Tul N et al. suggested that there is no difference in the level of serum inhibin A between pregnant women with hypertension during pregnancy and normal control group [9, 15], but Florio P et al. and Silver HM et al. suggested that inhibin A is associated with hypertension during pregnancy [2, 16]. Our results also suggest that serum inhibin A is an independent risk factor for preeclampsia and hypertensive disorder complicating pregnancy.

Insufficient invasion of maternal uterine spiral artery and placental dysfunction by trophoblast in preeclampsia are related to trophoblast dysfunction. Inhibin A, as a peptide hormone from placental trophoblast, can regulate embryo implantation differentiation and participate in placental maternal formation. The increase in synthetic ability is a compensatory response to the pathological process of the body, which can affect the release of placental hormones, stimulate the production of endothelin and thromboxane, lead to changes in the intrauterine environment, and cause abnormal phenotypic transformation of adhesion factors on the surface of trophoblast cells. It affects the reaction of cells and stroma and interferes with the infiltration of trophoblast cells. At the same time, inhibin A can affect the normal permeability and integrity of maternal blood vessels, affect the adaptability of maternal cardiovascular to pregnancy, reduce placental blood flow, aggravate placental ischemia and metabolic disorders, and lead to pathological changes of preeclampsia [20, 21].

With regard to the relationship between inhibin A and gestational diabetes mellitus, only one article has reported that the concentration of serum inhibin A in pregnant women with gestational diabetes mellitus is lower than that in the normal control group [15]. The study included only 27 patients with gestational diabetes, and our study included 1336 patients with gestational diabetes. There was no significant difference in the concentration of inhibin A between patients with gestational diabetes mellitus and normal controls. However, the level of serum inhibin A MoM in patients with gestational diabetes mellitus was significantly higher than that in the control group (1.13 ±0.54 MoM vs 1.07 ±0.47 MoM, *p* < 0.05). The results of regression analysis showed that inhibin A was also an independent risk factor for gestational diabetes mellitus. The presence of oxidative stress in the placenta of patients with GDM has been confirmed by recent studies. The study also suggests that changes in oxidative regulation balance in patients with GDM may be due to the placenta [22]. Morphological studies of placental structure during the occurrence of GDM suggest placental maturation and fibrinoid necrosis of local tissue [22]. Excessive formation of oxygen free radicals in gestational diabetes mellitus causes lipid peroxidation, damages the structure and function of placental tissue biofilm, and causes cell swelling and reactive proliferation. It can change the cell function, extracellular matrix and basement membrane of placenta, thus affecting the growth of placenta. GDM can be seen as a pathological disease of the placenta like preeclampsia. In gestational diabetes mellitus, the increase in inhibin A synthesis is a compensatory response to the pathological process of the placenta. For the first time, our study focused on the relationship between inhibin A and gestational diabetes mellitus, and found that the increase of inhibin A in the second trimester of pregnancy can be used as a risk factor for gestational diabetes mellitus.

Previous literature has only reported that high inhibin A concentration is associated with fetal growth restriction [2, 7, 14], but the relationship between inhibin A and abnormal neonatal weight has not been reported. Our results show that high levels of inhibin A are an independent risk factor for macrosomia and low birth weight infants, as far as I know, this is the first time that it has been reported. Maternal inhibin A with fetal growth restriction is similar to preeclampsia [23]. Decreased placental blood flow and insufficient fetal blood supply may be the factors leading to low birth weight infants, and the increase of inhibin A is a compensatory response to the pathological process of the body.

At present, there is no unified conclusion on the relationship between inhibin A and preterm delivery. Florio et al., Tul et al and Sehat et al. reported that high inhibin A levels are associated with the occurrence of preterm delivery [2, 15, 24], but Singnoi et al., Dugoff et al. and Beta et al. believed that there is no correlation between them [10, 17, 25]. Our results suggest that inhibin A is an independent risk factor for preterm delivery. The increase of inhibin-A in the second trimester of pregnancy may reflect the abnormality of placental development in the early stage of pregnancy and may cause obvious clinical manifestations in the third trimester of pregnancy. The unfavorable environment in the early stage of the uterus leads to the restriction of intrauterine growth of the fetus and leads to premature delivery.

Our large sample size reduces the selection bias that is prone to occurrence in retrospective case-control studies. Several studies have shown that inhibin A is not associated with gestational hypertension and preterm delivery, and the number of cases enrolled in these studies is smaller than that in our study, which may be one of the reasons for the differences. Another possible explanation for this difference is that other studies have used the concentration of inhibin A for risk factor analysis, and in our study we did not use the concentration of inhibin A directly. Instead, it is converted to a multiple of the median. During the conversion process, corrections were made for gestational weeks, body mass index, and insulin-dependent diabetes. Our research focuses on the predictive value of inhibin A in the second trimester of pregnancy for bad pregnancy, but the utility of assessing adverse pregnancy outcome risk using second trimester inhibinA is too late for aspirin in preventing preeclampsia. Serum inhibin A in early pregnancy needs to be further studied. In our group of adverse pregnancy outcomes, there was no distinction between macrosomia in the diabetic group and fetal growth restriction in the preeclampsia group. Therefore, for pregnant women with preeclampsia with fetal growth restriction and diabetes with macrosomia, the OR of inhibin A may be higher.

## Conclusion

The level of elevated Inhibin A in pregnancy is significantly related to the outcome of pre-eclampsia, GDM, macrosomia, low birth weight and preterm delivery. Clinicians should pay more attention to elevated inhibin A pregnant women and strengthen the supervision of pregnancy, which has important clinical significance to reduce pregnancy complications and avoid adverse pregnancy outcome.

## Supporting information

S1 Dataset (XLSX)

S1 Checklist STROBE statement—checklist of items that should be included in reports of observational studies. (DOCX)
