# Supplementary material for: Are serum levels of inhibin A in second trimester predictors of adverse pregnancy outcome?
Source: PLoS One. 2020 May 29;15(5):e0232634. doi: 10.1371/journal.pone.0232634 (PMC7259778; doi:10.1371/journal.pone.0232634)
Supplement: S1 Checklist — (DOCX) [file pone.0232634.s002.docx]

STROBE Statement—checklist of items that should be included in reports of observational studies

|  | Item No. | Recommendation | Page  No. | Relevant text from manuscript |
| --- | --- | --- | --- | --- |
| **Title and abstract** | 1 | (*a*) Indicate the study’s design with a commonly used term in the title or the abstract | 1 | Our aim was to assess whether second trimester serum inhibin A was associated with an increased risk of adverse pregnancy outcomes. |
|  |  | (*b*) Provide in the abstract an informative and balanced summary of what was done and what was found | 1 | Compared with the control group, during the second trimester of pregnancy, age and Inhibin A were risk factors for pre-eclampsia, gestational hypertension, gestational diabetes mellitus, low birth weight and preterm delivery; Gravidity and Inhibin A were risk factors for macrosomia; while parity was a protective factor against pre-eclampsia, gestational hypertension and low birth weight. |
| Introduction | | | |  |
| Background/rationale | 2 | Explain the scientific background and rationale for the investigation being reported | 2 | Previous studies have reported that the increase of inhibin A is associated with fetal malformation, fetal growth restriction and preeclampsia [2,6-10], but there are few reports on inhibin A and other maternal and fetal adverse pregnancy outcomes. |
| Objectives | 3 | State specific objectives, including any prespecified hypotheses | 2 | The purpose of our study was to evaluate the relationship between inhibition of A and preeclampsia, gestational hypertension, gestational diabetes, macrosomia, low birth weight and preterm delivery in Chinese pregnant women. |
| Methods | | | |  |
| Study design | 4 | Present key elements of study design early in the paper | 2 | We investigated the serum levels of Inhibin A during the second trimester in pregnancy, and analyzed associations between the Inhibin A and the risk of adverse pregnancy outcome. |
| Setting | 5 | Describe the setting, locations, and relevant dates, including periods of recruitment, exposure, follow-up, and data collection | 2-3 | A total of 12,124 singleton pregnancies women with a live delivery between January 2017 and July 2019 at the Obstetrics & Gynecology Hospital of Fudan University (Shanghai, China) were enrolled in this study. Participants included 8333 normal pregnancies in women and pregnancies in women with adverse pregnancy outcome were divided into six groups |
| Participants | 6 | (*a*) *Cohort study*—Give the eligibility criteria, and the sources and methods of selection of participants. Describe methods of follow-up  *Case-control study*—Give the eligibility criteria, and the sources and methods of case ascertainment and control selection. Give the rationale for the choice of cases and controls  *Cross-sectional study*—Give the eligibility criteria, and the sources and methods of selection of participants | 2-3 | Pre-eclampsia was diagnosed at weeks 34 through 39 of gestation, using the current American College of Obstetricians and Gynecologists (ASOG) guidelines [11]. Gestational hypertension is defined as diastolic blood pressure > 90 mmHg on two occasions four hours apart, or > 110 mmHg once, or systolic blood pressure > 140 mmHg on two occasions four hours apart, or > 160 mmHg once, after 20 weeks’ gestation (or a combination) [12]. GDM was diagnosed at 24–28 weeks of gestation using the American Diabetes Association (ADA) criteria [13]. Macrosomia was defined as birth weight >4000g. Low birth weight was defined as birth weight less than 2500 g. Preterm birth was defined as gestational age at birth of less than 37weeks.  Study exclusion criteria were as follows: established type 1 or type 2 diabetes; established hyperlipidemia, hypertension, cardiovascular diseases or metabolic syndrome before pregnancy; a history of severe systemic disease such as liver cirrhosis, chronic renal failure, severe anemia or immune disorders; and untreated endocrinopathies (hyperadrenalism, hypoadrenalism, and hyperthyroidism or hypothyroidism), or patient had no complete maternal and infant record. |
|  |  | (*b*) *Cohort study*—For matched studies, give matching criteria and number of exposed and unexposed  *Case-control study*—For matched studies, give matching criteria and the number of controls per case |  |  |
| Variables | 7 | Clearly define all outcomes, exposures, predictors, potential confounders, and effect modifiers. Give diagnostic criteria, if applicable | 3-4 | We measured the level of inhibin A in the serum of pregnant women, converted the measured value to a multiple of the median (MoM), and corrected it according to the mother's age, body mass, gestational age and whether there was insulin dependent diabetes mellitus.  with significant differences from the univariate analysis were included in the regression analysis. Multifactorial logistic regression analysis of each procedure was conducted based on univariate analyses. Regression analysis was used to adjust the influence of confounding factors, including age, parity, gravidity and pre-pregnancy BMI. |
| Data sources/ measurement | 8* | For each variable of interest, give sources of data and details of methods of assessment (measurement). Describe comparability of assessment methods if there is more than one group | *3* | The clinical data and outcomes for mothers and neonates were obtained from clinical records. All patient characteristics, including Inhibin A and Inhibin A MoM are described in Table 1 |
| Bias | 9 | Describe any efforts to address potential sources of bias | 6 | Our large sample size reduces the selection bias that is prone to occurrence in retrospective case-control studies. |
| Study size | 10 | Explain how the study size was arrived at | 2 | A total of 12,124 singleton pregnancies women with a live delivery between January 2017 and July 2019 at the Obstetrics & Gynecology Hospital of Fudan University (Shanghai, China) were enrolled in this study. |

Continued on next page

| Quantitative variables | 11 | Explain how quantitative variables were handled in the analyses. If applicable, describe which groupings were chosen and why | 3 | All statistical analyses were performed using GraphPad Prism version 5.0 for Windows (GraphPad Software, USA). Data are expressed as mean ± SD. Associations between Inhibin A and the risk of adverse pregnancy outcome were tested by multivariate logistic regression analysis. Variable selection in multivariable modeling was based on clinical and statistical significance. P < 0.05 was considered a statistically significant difference. |
| --- | --- | --- | --- | --- |
| Statistical methods | 12 | (*a*) Describe all statistical methods, including those used to control for confounding | 3 | All statistical analyses were performed using GraphPad Prism version 5.0 for Windows (GraphPad Software, USA). Data are expressed as mean ± SD. Associations between Inhibin A and the risk of adverse pregnancy outcome were tested by multivariate logistic regression analysis. Variable selection in multivariable modeling was based on clinical and statistical significance. P < 0.05 was considered a statistically significant difference. |
|  |  | (*b*) Describe any methods used to examine subgroups and interactions |  |  |
|  |  | (*c*) Explain how missing data were addressed | 3 | Study exclusion criteria were as follows: patient had no complete maternal and infant record. |
|  |  | (*d*) *Cohort study*—If applicable, explain how loss to follow-up was addressed  *Case-control study*—If applicable, explain how matching of cases and controls was addressed  *Cross-sectional study*—If applicable, describe analytical methods taking account of sampling strategy |  |  |
|  |  | (*e*) Describe any sensitivity analyses |  |  |
| Results | | | | |
| Participants | 13* | (a) Report numbers of individuals at each stage of study—eg numbers potentially eligible, examined for eligibility, confirmed eligible, included in the study, completing follow-up, and analysed | 2 | Participants included 8333 normal pregnancies in women and pregnancies in women with adverse pregnancy outcome were divided into six groups: pre-eclampsia (n=560), Gestational hypertension (n=505), gestational diabetes mellitus (GDM) (n=1336), macrosomia (n=699), low birth weight (n=276) and preterm delivery (n=415). |
|  |  | (b) Give reasons for non-participation at each stage |  |  |
|  |  | (c) Consider use of a flow diagram |  |  |
| Descriptive data | 14* | (a) Give characteristics of study participants (eg demographic, clinical, social) and information on exposures and potential confounders | 11 | Table 1. Characteristics of mothers and infants |
|  |  | (b) Indicate number of participants with missing data for each variable of interest | 2 | Participants included 8333 normal pregnancies in women and pregnancies in women with adverse pregnancy outcome were divided into six groups: pre-eclampsia (n=560), Gestational hypertension (n=505), gestational diabetes mellitus (GDM) (n=1336), macrosomia (n=699), low birth weight (n=276) and preterm delivery (n=415). |
|  |  | (c) *Cohort study*—Summarise follow-up time (eg, average and total amount) |  |  |
| Outcome data | 15* | *Cohort study*—Report numbers of outcome events or summary measures over time |  |  |
|  |  | *Case-control study—*Report numbers in each exposure category, or summary measures of exposure | *2* | Participants included 8333 normal pregnancies in women and pregnancies in women with adverse pregnancy outcome were divided into six groups: pre-eclampsia (n=560), Gestational hypertension (n=505), gestational diabetes mellitus (GDM) (n=1336), macrosomia (n=699), low birth weight (n=276) and preterm delivery (n=415). |
|  |  | *Cross-sectional study—*Report numbers of outcome events or summary measures |  |  |
| Main results | 16 | (*a*) Give unadjusted estimates and, if applicable, confounder-adjusted estimates and their precision (eg, 95% confidence interval). Make clear which confounders were adjusted for and why they were included | 12 | Table 2. Multivariate regression analysis of inhibin A and adverse pregnancy outcome |
|  |  | (*b*) Report category boundaries when continuous variables were categorized |  |  |
|  |  | (*c*) If relevant, consider translating estimates of relative risk into absolute risk for a meaningful time period |  |  |

Continued on next page

| Other analyses | 17 | Report other analyses done—eg analyses of subgroups and interactions, and sensitivity analyses |  |  |
| --- | --- | --- | --- | --- |
| Discussion | | | | |
| Key results | 18 | Summarise key results with reference to study objectives | 4 | Our study focused on serum inhibin A levels from 14 to 20 weeks of pregnancy and evaluated their relationship with these pregnancy complications. The results showed that serum inhibin A was an independent risk factor for preeclampsia, gestational hypertension, gestational diabetes, macrosomia, low birth weight and preterm delivery in Chinese pregnant women. |
| Limitations | 19 | Discuss limitations of the study, taking into account sources of potential bias or imprecision. Discuss both direction and magnitude of any potential bias | 6-7 | Our large sample size reduces the selection bias that is prone to occurrence in retrospective case-control studies. Several studies have shown that inhibin A is not associated with gestational hypertension and preterm delivery, and the number of cases enrolled in these studies is smaller than that in our study, which may be one of the reasons for the differences. Another possible explanation for this difference is that other studies have used the concentration of inhibin A for risk factor analysis, and in our study we did not use the concentration of inhibin A directly. Instead, it is converted to a multiple of the median. During the conversion process, corrections were made for gestational weeks, body mass index, and insulin-dependent diabetes. |
| Interpretation | 20 | Give a cautious overall interpretation of results considering objectives, limitations, multiplicity of analyses, results from similar studies, and other relevant evidence | 4-6 |  |
| Generalisability | 21 | Discuss the generalisability (external validity) of the study results | 4-6 |  |
| Other information | |  | | |
| Funding | 22 | Give the source of funding and the role of the funders for the present study and, if applicable, for the original study on which the present article is based | 7 | This work was supported by the program for National Natural Science Foundation of China (No. 81902131). |

*Give information separately for cases and controls in case-control studies and, if applicable, for exposed and unexposed groups in cohort and cross-sectional studies.

**Note:** An Explanation and Elaboration article discusses each checklist item and gives methodological background and published examples of transparent reporting. The STROBE checklist is best used in conjunction with this article (freely available on the Web sites of PLoS Medicine at http://www.plosmedicine.org/, Annals of Internal Medicine at http://www.annals.org/, and Epidemiology at http://www.epidem.com/). Information on the STROBE Initiative is available at www.strobe-statement.org.
